# Thiosulfate Transfer Mediated by DsrE/TusA Homologs from Acidothermophilic Sulfur-oxidizing Archaeon Metallosphaera cuprina*

**DOI:** 10.1074/jbc.M114.591669

**Published:** 2014-08-13

**Authors:** Li-Jun Liu, Yvonne Stockdreher, Tobias Koch, Shu-Tao Sun, Zheng Fan, Michaele Josten, Hans-Georg Sahl, Qian Wang, Yuan-Ming Luo, Shuang-Jiang Liu, Christiane Dahl, Cheng-Ying Jiang

**Affiliations:** From the ‡ State Key Laboratory of Microbial Resources, Institute of Microbiology, Chinese Academy of Sciences, Beijing 100101, China,; the ¶ Institut für Mikrobiologie & Biotechnologie, Rheinische Friedrich-Wilhems-Universität Bonn, 53115 Bonn, Germany,; the ‖ Core Facility and; ‡‡ Environmental Microbiology Research Center, Institute of Microbiology, Chinese Academy of Sciences, Beijing 100101, China,; the § University of Chinese Academy of Sciences, Beijing 100049, China, and; the ** Institut für Medizinische Mikrobiologie, Immunologie und Parasitologie, Abteilung Pharmazeutische Mikrobiologie, Rheinische Friedrich-Wilhelms-Universität Bonn, 53127 Bonn, Germany

**Keywords:** Archaea, Energy Metabolism, Microbiology, Oxidation-Reduction (Redox), Sulfur, DsrE/TusA-like Proteins, *Metallosphaera cuprina*, Thiosulfate Transfer, Tetrathionate

## Abstract

Conserved clusters of genes encoding DsrE and TusA homologs occur in many archaeal and bacterial sulfur oxidizers. TusA has a well documented function as a sulfurtransferase in tRNA modification and molybdenum cofactor biosynthesis in *Escherichia coli*, and DsrE is an active site subunit of the DsrEFH complex that is essential for sulfur trafficking in the phototrophic sulfur-oxidizing *Allochromatium vinosum*. In the acidothermophilic sulfur (S^0^)- and tetrathionate (S_4_O_6_^2−^)-oxidizing *Metallosphaera cuprina* Ar-4, a *dsrE3A-dsrE2B-tusA* arrangement is situated immediately between genes encoding dihydrolipoamide dehydrogenase and a heterodisulfide reductase-like complex. In this study, the biochemical features and sulfur transferring abilities of the DsrE2B, DsrE3A, and TusA proteins were investigated. DsrE3A and TusA proved to react with tetrathionate but not with NaSH, glutathione persulfide, polysulfide, thiosulfate, or sulfite. The products were identified as protein-Cys-*S*-thiosulfonates. DsrE3A was also able to cleave the thiosulfate group from TusA-Cys^18^-*S*-thiosulfonate. DsrE2B did not react with any of the sulfur compounds tested. DsrE3A and TusA interacted physically with each other and formed a heterocomplex. The cysteine residue (Cys^18^) of TusA is crucial for this interaction. The single cysteine mutants DsrE3A-C^93^S and DsrE3A-C^101^S retained the ability to transfer the thiosulfonate group to TusA. TusA-C^18^S neither reacted with tetrathionate nor was it loaded with thiosulfate with DsrE3A-Cys-*S*-thiosulfonate as the donor. The transfer of thiosulfate, mediated by a DsrE-like protein and TusA, is unprecedented not only in *M. cuprina* but also in other sulfur-oxidizing prokaryotes. The results of this study provide new knowledge on oxidative microbial sulfur metabolism.

## Teaser

**Background:** The *dsrE3A-dsrE2B-tusA* cluster from *Metallosphaera cuprina* encodes putative sulfur-trafficking proteins and is flanked by genes encoding a heterodisulfide reductase-like complex and dihydrolipoamide dehydrogenase.

**Results:** DsrE3A and TusA react with tetrathionate, yielding protein Cys-S-thiosulfonates, triggering irreversible thiosulfate transfer from DsrE3A to TusA.

**Conclusion:** DsrE3A and TusA are important players during dissimilatory sulfur and tetrathionate oxidation.

**Significance:** Protein-mediated transfer of thiosulfonate is unprecedented in sulfur oxidizers.

## Introduction

Elemental sulfur (S^0^) and reduced inorganic sulfur compounds serve as energy sources and electron donors for a number of chemo- and photolithotrophic bacteria such as *Acidithiobacillus* species (1–3) and *Allochromatium vinosum* (4). Dissimilatory sulfur oxidation also occurs in the archaeal domain of prokaryotes and is well known for chemolithotrophic acidophiles such as *Sulfolobus*, *Acidianus*, and *Metallosphaera*. Species of the genus *Metallosphaera* typically grow by aerobic respiration on CO_2_ with S^0^, pyrite, and tetrathionate (S_4_O_6_^2−^) as electron donors (5, 6). The best characterized archaeal enzyme involved in sulfur oxidation is probably sulfur oxygenase reductase, identified in *Acidianus* and present also in some *Sulfolobus* species. *In vitro* the enzyme catalyzes disproportionation of S^0^ into sulfide, sulfite, and thiosulfate (7–9). Sulfur oxygenase reductase is not present in *Metallosphaera* (10, 11).

Thiosulfate and tetrathionate are important intermediates that play key roles during sulfur oxidation by bacteria and archaea. Although the periplasmic Sox multienzyme for thiosulfate degradation is widespread in bacterial sulfur oxidizers, it is not found in acidophilic sulfur-oxidizing archaea (12, 13). Instead, in organisms such as *Acidianus ambivalens* two thiosulfate molecules are oxidatively condensed to tetrathionate in a reaction catalyzed by the membrane-bound cytoplasmically oriented thiosulfate:quinone oxidoreductase (TQO)^5^ (14). Although TQO is also present in a few bacteria, the main catalyst of tetrathionate formation in the Bacteria domain appears to be the soluble, periplasmic *c*-type cytochrome TsdA (15, 16). Sulfide:Quinone oxidoreductase is a widespread sulfide-oxidizing enzyme not only in bacteria but also in archaeal sulfur oxidizers like *Metallosphaera cuprina* (11). In the genera *Acidianus* and *Metallosphaera*, electrons from sulfide as well as from thiosulfate are thus fed into the quinone pool and coupled to ATP generation via oxidative phosphorylation (17).

**FIGURE 1.**
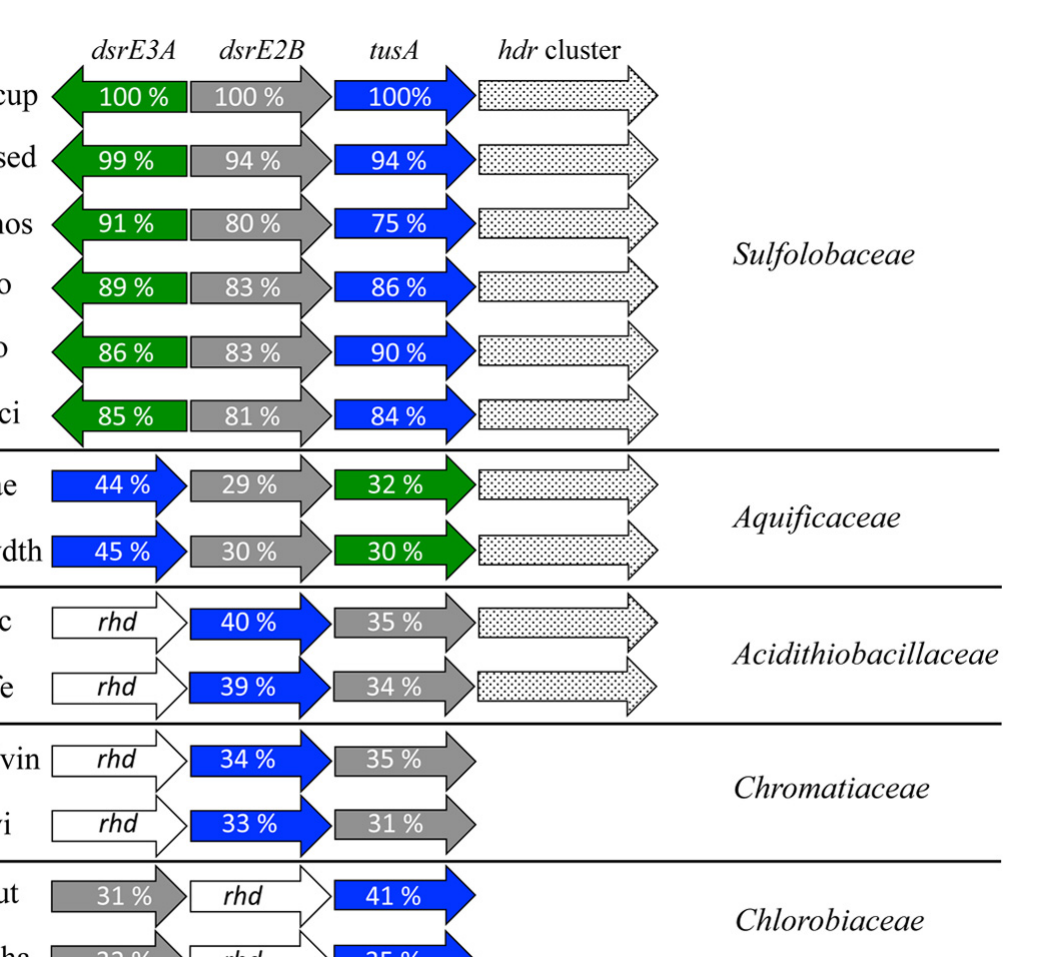
**Organization of *dsrE-tusA*-like genetic clusters in diverse archaeal and bacterial groups.** Amino acid sequence identities (*numbers* are shown in each gene) of DsrE-like and TusA-like proteins. Different genes are represented by *colors*. Mcup, *Metallosphaera cuprina* Ar-4 (NC_015435); Msed, *Metallosphaera sedula* DSM 5348 (NC_009440); Ahos, *Acidianus hospitalis* W1 (NC_015518); Sso, *Sulfolobus solfataricus* P2 (NC_002754); Sto, *Sulfolobus tokodaii* str.7 (NC_003106); Saci, *Sulfolobus acidocaldarius* DSM 639 (NC_007181); Aae, *Aquifex aeolicus* VF5 (NC_000918); Hydth, *Hydrogenobacter thermophilus* TK-6 (NC_017161); Atc, *Acidithiobacillus caldus* SM-1 (NC_015850); Afe, *Acidithiobacillus ferrooxidans* ATCC 23270 (NC_011761); Alvin, *Allochromatium vinosum* DSM 180 (NC_013851); Tvi, *Thiocystis violascens* DSM 198 (NC_018012); Plut, *Chlorobium luteolum* DSM 273 (NC_007512); Ppha, *Pelodictyon phaeoclathratiforme* BU-1 (NC_011060).

Many sulfur-oxidizing bacteria form conspicuous sulfur globules as intermediates during the oxidation of sulfide, polysulfides, or thiosulfate. The sulfur globules are deposited either extracellularly or intracellularly in the periplasm (*e.g.* in *Allochromatium* (18) or *Beggiatoa* (19) species). In *A. vinosum*, the degradation of the sulfur globules involves essential steps in the cytoplasm and is catalyzed by soluble and membrane-bound proteins of the Dsr system (18, 20–22). It is well established that the Dsr mechanism involves transport of sulfur into the cytoplasm and an extensive sulfur trafficking network. DsrC is the final sulfur-accepting protein, and in its persulfurated form it serves as a direct substrate for dissimilatory sulfite reductase (DsrAB), the enzyme that catalyzes the formation of sulfite. DsrC receives sulfur from DsrEFH, which in turn is sulfurated by TusA. Sulfane sulfur is mobilized from low molecular weight persulfides and transferred to TusA by a rhodanese-like protein. Furthermore, the whole process possibly involves a DsrE-like protein, termed DsrE2, encoded in the same gene cluster (*rhd-tusA-dsrE2*) (23, 24). Notably, an *rhd-tusA-dsrE2* or at least a *tusA-dsrE2* arrangement also occurs in many photo- and chemotrophic sulfur oxidizers that do not contain DsrC and the Dsr pathway (25, 46). Those sulfur oxidizers include archaeal sulfur oxidizers such as *Acidianus hospitalis* (26), *Sulfolobus tokodaii* (27), *Metallosphaera sedula* (28), and *M. cuprina* (11), as well as bacterial sulfur oxidizers such as members of the family Aquificaceae (29, 30) and the genera *Acidithiobacillus* (31) and *Thioalkalivibrio* (32) (Fig. 1). Inevitably, in this group the putative *tu*sA-*dsrE*2 genes are linked with the gene cluster *hdrC1B1AhyphdrC2B2* that encodes a possible heterodisulfide-reductase complex. This complex has been predicted to be responsible for the oxidation of organic persulfides to sulfite in *Acidithiobacillus ferrooxidans* based on the observation that the *tusA-dsrE2-hdr* genes were transcriptionally up-regulated when elemental sulfur was utilized as energy source (25). Additionally, transcription of *dsrE2*-, *tusA*-, or *hdr*-like gene was also up-regulated in *M. sedula* when S^0^ or tetrathionate was provided as an electron donor (28).

This study aimed at gaining information about the function and biochemical properties of DsrE- and TusA-like proteins in the acidothermophilic archaeon *M. cuprina* (6). To this end, a bioinformatics approach was combined with *in vitro* studies that demonstrated not only tight interaction but also transfer of thiosulfate between archaeal TusA and one of the DsrE homologs.

## EXPERIMENTAL PROCEDURES

### 

#### 

##### Strains, Plasmids, Media, and Growth Conditions

Bacterial strains and plasmids used in this study are listed in Table 1. *M. cuprina* Ar-4 was cultivated at 65 °C and pH 3.0 in modified Allen medium with 1 g/liter yeast extract (6, 33). *Escherichia coli* DH5α was used for molecular cloning of genes (Table 1). *E. coli* BL21(DE3)/pLysRARE was used for overproduction of recombinant proteins. All *E. coli* strains were grown in Luria-Bertani medium at 37 °C. The antibiotics used were at 100 μg/ml for ampicillin and 25 μg/ml for chloramphenicol.

##### Genetic Cloning and Site-directed Mutagenesis

The targeted genes were PCR-amplified with *Pfu* DNA polymerase using genomic DNA of *M. cuprina* Ar-4 as template. Primers are listed in Table 1. The PCR product was purified after digestion and was cloned into vectors pET15b (Novagen, Darmstadt, Germany) and pPRIBA1 (IBA GmbH, Göttingen, Germany). Positive recombinant plasmids were selected and verified by nucleotide sequencing. Plasmids used in this study are listed in Table 1.

Replacements of cysteine residue by serine residue (site-directed mutations) were performed with gene splicing by overlap extension (34). Plasmids carrying targeted genes were used as PCR template. Forward and reverse primers (Table 1) complementary to the plasmid sequence (pET15b or pPRIBA1) were 700–1000 bp upstream or downstream from the target gene. The double mutant *dsrE3A*-C^93^S/C^101^S was constructed by introducing the C^101^S mutation to pET15b-*dsrE3A*-C^93^S. All of the genetic constructions were sequenced to exclude any PCR amplification errors.

**TABLE 1:** **Archaeal and bacterial strains, plasmids, and primers used in this study** Sites that recognize restriction enzyme are underlined. The italic nucleotides are codons for serine.

Strain, plasmid, or primer	Description	Reference or source
**Strains**		
*E. coli* DH5α	F^−^ ϕ80d *lacZ*ΔM15Δ (*lacZYA-argF*)U169 *recA1endA1 hsdR17* (r_K_^−^m_K_^+^) *supE44* λ^[minus^*^]^ thi-1 gyrA relA1*	Ref. 49
*E. coli* BL21(DE3)	F^−^ *ompT hsd*S_B_ (r_B_^−^ m_B_^−^) *gal dcm* (DE3)	Novagen
*M. cuprina* Ar-4	JCM 15769^T^	Ref. 6

**Plasmids**		
pET15-*dsrE3A*	Amp^r^, T7 promoter, N-terminal His tag, XhoI/BamHI fragment of amplified *dsrE3A*	This work
pET15-*dsrE3A-*C^93^S	Amp^r^, T7 promoter, N-terminal His tag, XhoI/BamHI fragment of amplified *dsrE3A-*C^93^S	This work
pET15-*dsrE3A*-C^101^S	Amp^r^, T7 promoter, N-terminal His tag, XhoI/BamHI fragment of amplified *dsrE3A-*C^101^S	This work
pET15-*dsrE3A*-C^93^S/C^101^S	Amp^r^, T7 promoter, N-terminal His tag, XhoI/BamHI fragment of amplified *dsrE3A-*C^93^S/C^101^S	This work
pET15-*dsrE*2B	Amp^r^, T7 promoter, N-terminal His tag, NdeI/BamHI fragment of amplified *dsrE2B*	This work
pET15-*dsrE*2B-C^99^S	Amp^r^, T7 promoter, N-terminal His tag, NdeI/BamHI fragment of amplified *dsrE2B-*C^99^S	This work
pET15-TusA	Amp^r^, T7 promoter, N-terminal His tag, NdeI/BamHI fragment of amplified *tusA*	This work
pET15-TusA-C^18^S	Amp^r^, T7 promoter, N-terminal His tag, NdeI/BamHI fragment of amplified *tusA*-C^18^S	This work
pPRIBA1-TusA	Amp^r^, T7 promoter, C-terminal Strep-tag, NdeI/Eco47III fragment of amplified *tusA*	This work

**Primers**		
15b-*3A*-for	TGAGGGCTCGAGATGGCACAAACC	This work
15b-*3A*-rev	CAAATTGGATCCGTATCAGAAGAACAG	This work
15b-*2B*-for	GATATACTGGTGAACATATGGCAGG	This work
15b-*2B*-rev	CGTAGGATCCTCAAATGAACAGAG	This work
15b-*tusA*-for	ATACATATGGCTCAAGATGTAAAG	This work
15b-*tusA*-rev	TAAAATAGGATCCGTTACTTCGCTCTC	This work
Strep-*tusA*-rev	TAAAATAGCGCTCTTCGCTCTCTTCAC	This work
C^93^S-for	GATGTACGTA*AGT*GTACAAAG	This work
C^93^S-rev	CTTTGTACACTTACGTACATC	This work
C^101^S-for	GGACATG*AGT*CATATGAACG	This work
C^101^S-rev	CGTTCATATGACTCATGTCC	This work
C^99^S-for	TCTACGCT*AGC*TCAACCAC	This work
C^99^S-rev	GTGGTTGAGCTAGCGTAGA	This work
C^18^S-for	GGAATGTAT*AGT*CCAGGTCC	This work
C^18^S-rev	GGACCTGGACTATACATTCC	This work
pET15b-for	GACTGGAGGTGGCAACGC	This work
pET15b-rev	CTCTCAAGGATCTTACCGC	This work
pPRIBA1-for	CCTCGCTCTGCTAATCCTG	This work
pPRIBA1-rev	GCCACCTAAATTGTAAGCG	This work

##### Molecular Evolutionary Analysis

A molecular evolutionary analysis was performed using MEGA6 software (35). The phylogeny test was executed with UPGMA (unweighted pair group method with arithmetic mean) (36), a statistical method that applies a bootstrap test with 10,000 replicates (37). The evolutionary distances were computed using the Poisson correction method (38).

##### Overproduction and Purification of the Recombinant Proteins

Recombinant DsrE2B, DsrE3A, and TusA were produced in *E. coli* BL21(DE3)/pLysRARE. Overnight precultures were used to inoculate fresh LB medium with a ratio of 1:60 (v/v). Synthesis of recombinant proteins in cells was induced by the addition of 0.1 mm IPTG when the culture reached an OD_600_ of 0.6–0.8 and was further incubated for 2.5 h at 37 °C before harvesting. Cells were pelleted at 3000 × *g* for 10 min, and were resuspended in buffer containing 50 mm Tris-HCl and 150 mm NaCl (pH 7.5). Cells were broken by sonification. Cellular lysate was centrifuged at 17,000 × *g* for 20 min at 4 °C. The supernatant was incubated in a water bath at 65 °C for 10 min, and the denatured proteins were removed by centrifugation at 17,000 × *g* for 20 min. The supernatant containing the recombinant protein was filtered with a 0.45-μm filter membrane (Millipore, Darmstadt, Germany) before being loaded to the gravity flow column for purification. His-tagged and Strep-tagged proteins were purified with TALON metal affinity resin (Clontech) and Strep-Tactin Superflow (IBA), respectively, according to protocols provided by the suppliers.

##### Visualization of Cysteine Modifications by N-(Iodoacetyl)-N′-(5-sulfo-1-naphthyl)ethylenediamine (1,5-I-AEDANS) Gel Assays

The principle of 1,5-I-AEDANS gel assays has been described by Zheng *et al.* (39). Protein was treated with 5 mm DTT at 37 °C for 20 min to break down any disulfide bonds and to release cysteine residues. DTT was then removed by using PD MiniTrap G-25 columns (GE Healthcare). In a typical assay, each protein was incubated at 65 °C for 30 min with 5 mm substrate in buffer (pH 7.5) containing 50 mm Tris-HCl and 150 mm NaCl. In parallel, a reference test was run at identical conditions but without substrate. Excessive substrate was removed by using PD MiniTrap G-25 columns. Six substrates were tested in this study: sodium hydrosulfide (NaSH), glutathione persulfide (GSSH), polysulfide (S_*n*_^2−^), thiosulfate (S_2_O_3_^2−^), tetrathionate (S_4_O_6_^2−^), and sulfite (SO_3_^2−^). GSSH was synthesized according to the method of Rohwerder and Sand (40) as specified in Stockdreher *et al.* (24). Proteins were concentrated to ∼0.4 mm with Sartorius Vivaspin 500 centrifugal concentrators (5000 Da).

Visualization of cysteine modification of cystein residues by 1,5-I-AEDANS proceeded as follows. 5–10 μl of the concentrated sample was treated with 3 μl of 2 mm 1,5-I-AEDANS at 4 °C for at least 1 h before adding 2 μl of 8 mm l-cysteine at room temperature for 30 min to react with the excessive 1,5-I-AEDANS. 1 μl of 100 mm DTT was added to the reaction mixture as described (41). Our results indicated that treatment with DTT did not affect reaction of proteins with 1,5-I-AEDANS. Native loading buffer was applied to the sample followed by electrophoresis on 15% Tris-glycine SDS-polyacrylamide gels in the dark. The fluorescence of 1,5-AEDANS was detected under UV light, and proteins were stained with Coomassie Brilliant Blue R-250.

##### Determination of Thiosulfate Transfer

In a typical thiosulfate transfer assay, 1.5 nmol of thiosulfate donor proteins (DsrE3A or TusA after reaction with substrate) was incubated with equal amounts of thiosulfate acceptor proteins (TusA or DsrE3A) at 65 °C for 30 min. Thiosulfate transfer was evaluated by determination of cysteine modification, and visualization of cysteine modification was carried out as described above.

##### MALDI-TOF Mass Spectrometry

For MALDI-TOF MS, the matrix was sinapinic acid in 50% acetonitrile and 0.1% trifluoroacetic acid solution. The buffer of protein samples was exchanged for 0.1% trifluoroacetic acid by using a PD MiniTrap G-25 column. About 10-pmol samples were detected in the positive linear mode with a Biflex III (Bruker Daltonics GmbH, Leipzig, Germany) or AB SCIEX TOF/TOF 5800 (AB SCIEX, Framingham, MA).

##### Strep-tag® Pulldown Assay

During the Strep-tag pulldown assay, 10 nmol of Strep-tagged proteins and 30 nmol of non-Strep-tagged proteins were incubated together with 0.75 ml of Strep-Tactin Superflow on ice for 1 h. The mixture was then loaded to the gravity flow column to continue the pulldown assay. Reference tests were run in parallel under identical conditions, but only the non-Strep-tagged protein was incubated with Strep-Tactin Superflow.

##### Surface Plasmon Resonance

A Biacore 3000 instrument (GE Healthcare) was equipped with a CM5 sensor chip (GE Healthcare) at 25 °C. DsrE3A (26 μg/ml) proteins (ligand) in 10 mm acetic acid (pH 5.5) were covalently immobilized to the chip according to the protocol provided by the supplier, and the resonance units reached about 1500. PBST buffer (PBS containing 0.005% Tween 20 (pH 7.4)) was used as the running buffer with a flow rate of 30 μl/min. TusA proteins (analyte) in PBST buffer was injected for 2 min at a flow rate of 30 μl/min. Dissociation of protein-protein complexes on the sensor surface proceeded for 8 min by flowing PBST buffer over the sensor surface. A blank injection with PBST buffer was used as the control. The surface was regenerated with 5–60 μl of 10 or 20 mm NaOH. A control surface without ligand was used as a reference.

##### Gel Filtration

DsrE3A and TusA were incubated on ice for at least 1 h before being injected with a 2-ml sample loop to AKTA^TM^ purifier equipped with a HiLoad 16/60 Superdex 200 prep grade column (GE Healthcare). A solution of 50 mm Tris-HCl (pH 7.5) and 150 mm NaCl was used as the running buffer, and the flow rate was kept constant at 1.0 ml/min. Proteins were incubated with or without 25 μm Tris(2-carboxyethyl)phosphine (TCEP) at room temperature for 1 h before injection to the AKTA^TM^ purifier with a 0.5-ml sample loop for oligomerization analysis. The column used in these cases was a HiLoad 16/60 Superdex 75 prep grade column (GE Healthcare). 50 mm Tris-HCl (pH 7.5) and 150 mm KCl was used as the running buffer with a flow rate 0.5 ml/min. Blue dextran (2000 kDa), bovine catalase (240 kDa), bovine albumin (67 kDa), egg albumin (45 kDa), chymotrypsinogen A (25 kDa), equine myoglobin (17 kDa), and cytochrome *c* (12.3 kDa) were used for calibration.

## RESULTS

### 

#### 

##### Occurrence, Genetic Environment, and Grouping of Archaeal DsrE Proteins

**FIGURE 2.**
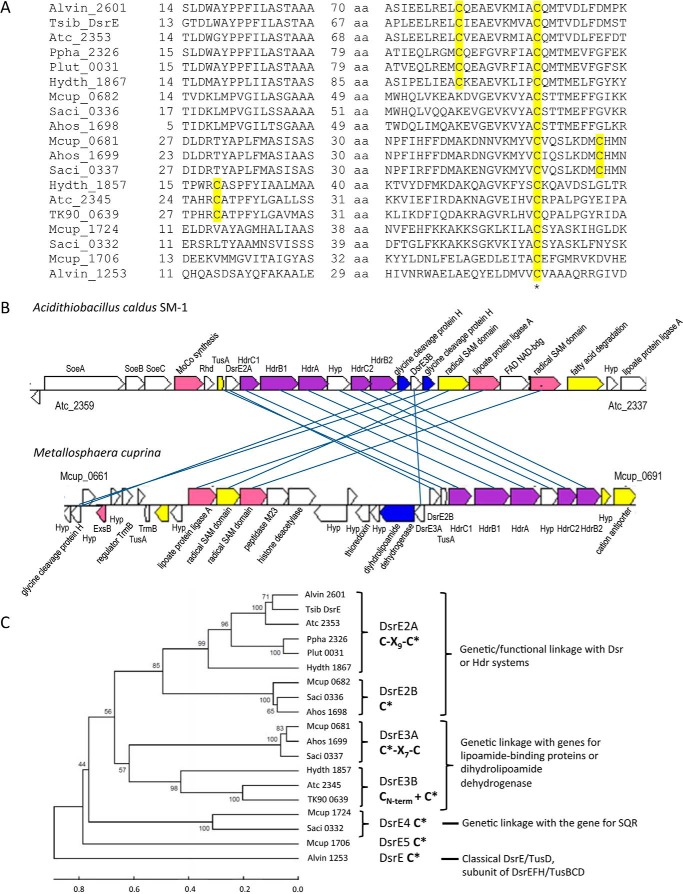
**Alignment of DsrE homologous sequences (*A*), comparison of gene clusters containing *dsrE*, *tusA*, and *hdr* genes in *A. caldus* SM-1 and *M. cuprina* Ar-4 (*B*), and phylogenetic tree for DsrE orthologs from the Bacteria and Archaea domains (*C*).** The DsrE homologous sequences in *A* are from the following orders: Chromatiales: *A. vinosum* DSM 180 (Alvin), *Thiorhodospira sibirica* ATCC 700588 (Tsib), and *Thioalkalivibrio* sp. K90mix (TK90); Acidithiobacillales: *A. caldus* SM-1 (Atc); Chlorobiales: *C. luteolum* DSM 273 (Plut) and *P. phaeoclathratiforme* BU-1 (Ppha); Aquificales: *H. thermophilus* TK-6 (Hydth); and Sulfolobales: *M. cuprina* Ar-4 (Mcup), *A. hospitalis* W1 (Ahos), and *S. acidocaldarius* DSM 639 (Saci). The sequences are identified by their locus tags. The active site cysteine conserved in all sequences is labeled with an *asterisk*. For clarity, only those parts of the alignments are shown that contain conserved cysteine residues. The complete alignment served as the basis for the tree shown in *C*. In *C*, the evolutionary history was inferred using the UPGMA method. The optimal tree with the sum of branch length = 7.16951712 is shown. The percentages of replicate trees in which the associated taxa clustered together in the bootstrap test (10,000 replicates) are shown next to the branches. The tree is drawn to scale, with branch lengths in the same units as those of the evolutionary distances used to infer the phylogenetic tree. The evolutionary distances were computed using the Poisson correction method and are in the units of the number of amino acid substitutions per site. The analysis involved the 19 amino acid sequences aligned in *A*.

The genome of *M. cuprina* encodes four different DsrE-like proteins: Mcup_0681, Mcup_0682, Mcup_1706, and Mcup_1724. All of these proteins contain cysteine residues corresponding to the active cysteine residue of DsrE from *A. vinosum* (Fig. 2*A*), which had been demonstrated experimentally (23). With the exception of only Mcup_0681, the *M. cuprina dsrE*-like genes reside close to genes encoding TusA homologs. All of the archaeal *dsrE*-like genes reside amid genes connected to oxidative sulfur metabolism. Mcup_1706 and Mcup_1724 are part of a “sulfur island” comprising TQO genes (Mcup_1712 and Mcup_1713) and a gene for a protein with a sulfate transporter domain, as well as the gene for a potential sulfide:quinone oxidoreductase (Mcup_1723) and TusA (Mcup_1722). Mcup_0682 and a further *tusA* homolog (Mcup_0683) immediately precede the *hdrC1B1AhyphdrC2B2* cluster (Fig. 2*B*). Mcup_0681 is transcribed in the opposite direction to Mcup_0682 and resides upstream of a genetic cluster that encodes a putative dihydrolipoamide dehydrogenase (Mcup_0680), a hypothetical protein (Mcup_0679), and a putative thioredoxin (Mcup_0678). Notably, a lipoamide-binding protein resembling protein H of the glycine cleavage system (Mcup_0662) and several proteins responsible for the biosynthesis of lipoamide-containing proteins (Mcup_0671–0673) are also encoded in the vicinity of these genes (Fig. 2*B*). In fact, related genes are also part of or reside in the immediate vicinity of all the *tusA-dsrE-hdr* genetic clusters in other genome-sequenced archaeal and bacterial sulfur oxidizers. As an example, the organization of the respective genes in *Acidithiobacillus caldus* is compared with that in *M. cuprina* in Fig. 2*B*.

Sequence alignments and phylogenetic analyses provided us with the basis for grouping DsrE homologs into the following five categories (Fig. 2*C*). 1) The DsrE group consists of subunits of DsrEFH (prototype Alvin_1253). 2) The DsrE2 group consists of members that are genetically or functionally linked with Dsr or Hdr systems. Subgroup DsrE2A (prototype Alvin_2601) contains two strictly conserved cysteines. The prototype of subgroup DsrE2B is Mcup_0682. 3) The genes of the DsrE3 group are either immediately linked with genes for dihydrolipoamide dehydrogenase (DsrE3A, prototype Mcup_0681) or with genes for lipoamide-binding proteins (DsrE3B, prototype Atc_2345). The proteins of group DsrE3A contain two conserved cysteine residues in a Cys-*X*_7_-Cys motif with the first cysteine corresponding to the established DsrE active site cysteine (Fig. 2*A*). 4) The members of the DsrE4 group are encoded downstream of sulfide:quinone oxidoreductase (prototype Mcup_1724). 5) Group DsrE5 is represented by Mcup_1706.

Similar genetic organizations (*i.e. dsrE-tusA*) were observed in sulfur-oxidizing species of the families Sulfolobaceae, Aquificaceae, Acidithiobacillaceae, Chromatiaceae, and Chlorobiaceae (Fig. 1). It appears that the *dsrE* gene might have been duplicated (as *dsrE3A* and *dsrE2B*) in members of the Sulfolobaceae and Aquificaceae. Genes encoding proteins or enzymes that are involved in reversible reduction of heterodisulfide bonds coupled with energy conservation (Hdr complex) or in sulfur oxidation (rhodanese (Rdh)-like protein) were found in conjunction with the *dsrE-tusA* cluster as shown in Fig. 1. The DsrE3A of *M. cuprina* Ar-4 had 24% (89% coverage) and 41% (47% coverage) identity to the DsrE2A (AFE_2556) of *A. ferrooxidans* and DsrE2A (Alvin_2601) of *A. vinosum*, respectively. DsrE2B of *M. cuprina* Ar-4 had 34% (100% coverage) and 35% (86% coverage) identity to AFE_2556 and Alvin_2601, respectively. TusA (Mcup_0683) of *M. cuprina* Ar-4 has 39% (88% coverage) and 34% (91% coverage) identity to TusA (AFE_2557) of *A. ferrooxidans* and TusA (Alvin_2600) of *A. vinosum*, respectively.

##### Cloning, Site-directed Mutagenesis, and Expression of dsrE2B, dsrE3A, and tusA from M. cuprina in E. coli

N-terminally His-tagged DsrE3A (Mcup_0681), DsrE2B (Mcup_0682), and TusA (Mcup_0683) as well as TusA carrying a carboxyl-terminal Strep-tag were produced in *E. coli* BL21(DE3)/pLysRARE and purified by affinity chromatography. In all cases, the apparent molecular masses matched the theoretical masses for DsrE3A, DsrE2B, and TusA (His-tagged) at 17,697, 17,480, and 10,895 Da, respectively, with TusA Strep-tagged at 9,930 Da. In addition, six mutants (DsrE3A-C^93^S, DsrE3A-C^101^S, DsrE3A-C^93^S/C^101^S, DsrE2B-C^99^S, and TusA-C^18^S with His-tags and TusA-C^18^S with a Strep-tag) were produced, of which potentially important cysteine residues were replaced by serine residues.

The purified DsrE3A molecules as well as all derivatives occurred in homotrimers as indicated by gel filtration. It was not clear whether DsrE2B and DsrE2B-C^99^S were homodimers or homotrimers (data not shown). The oligomerization of DsrE3A and DsrE2B as trimers matches the structures deposited in the Protein Data Bank (PDB) for the corresponding proteins from *Sulfolobus solfataricus* P2 (DsrE3A: SSO1125, PDB ID 3MC3; DsrE2B: SSO1126, PDB ID 2QS7). TusA of *M. cuprina* occurred both as monomers and dimers. The mutant protein TusA-C^18^S occurred only in the monomeric state, suggesting that dimer formation depended on the presence of the Cys^18^ residue of wild-type TusA (data not shown).

##### DsrE3A and TusA React with Tetrathionate and Form Protein-Cys-S-thiosulfonate

**FIGURE 3.**
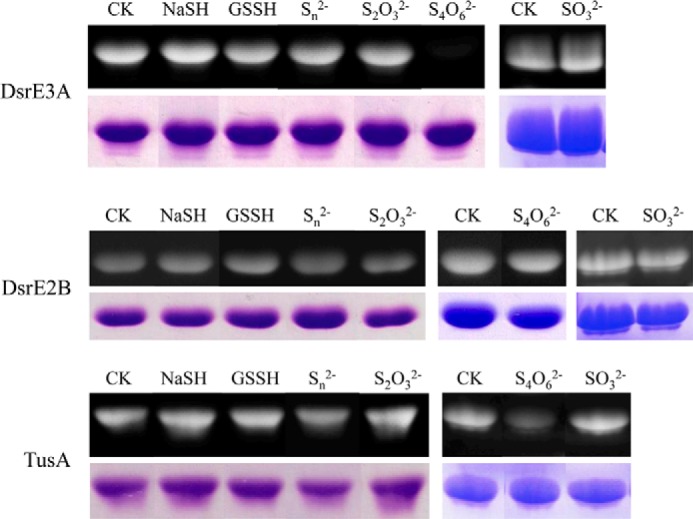
**Detection for DsrE3A, DsrE2B, and TusA modification by NaSH, GSSH, polysulfide (S_*n*_^2−^), thiosulfate (S_2_O_3_^2−^), tetrathionate (S_4_O_6_^2−^), and sulfite (SO_3_^2−^) by 1,5-I-AEDANS gel assays.** *CK*, without any substrates. The gels were exposed to UV light (*upper panel* for each protein) or stained with Coomassie Brilliant Blue (*lower panel* for each protein).

**FIGURE 4.**
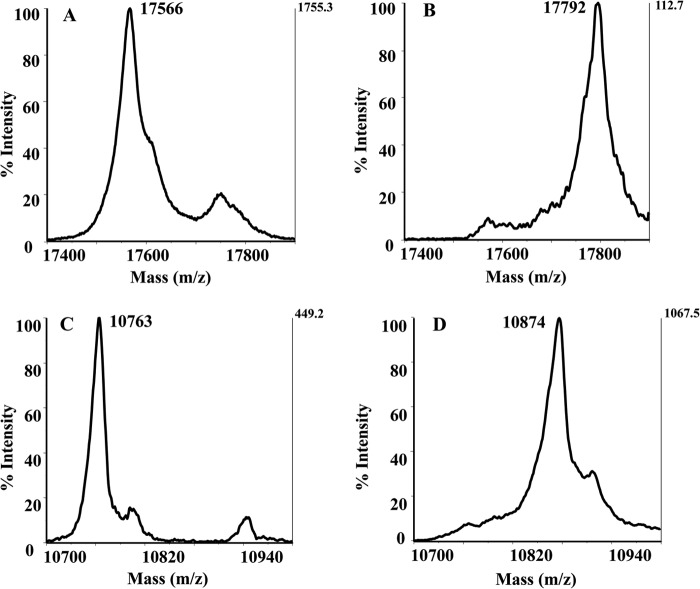
**Detection for DsrE3A (*A* and *B*) and TusA (*C* and *D*) modification by tetrathionate with MALDI-TOF mass spectrometry.** DsrE3A (*B*) and TusA (*D*) were modified by thiosulfate group(s) after reaction with tetrathionate. The observed protein masses without any modifications are 131 Da smaller than the corresponding theoretical masses. This is due to the cleavage of the first methionine residue when it precedes glycine residues (47, 48). The 4-Da difference was attributed to measurement error in the positive linear mode by the MALDI-TOF MS instruments.

**SCHEME 1:**
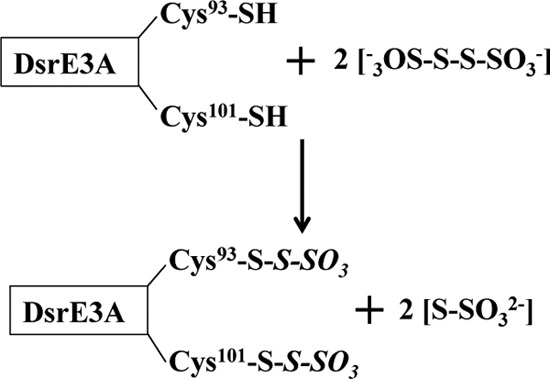


To investigate their functions, DsrE3A (Mcup_0681), DsrE2B (Mcup_0682), and TusA (Mcup_0683) were incubated separately with six different sulfur-containing compounds: NaSH, GSSH, polysulfide, thiosulfate, tetrathionate, and sulfite. The untreated proteins and the proteins treated with sulfur-containing compounds were reacted with the fluorescent thiol-reactive reagent 1,5-I-AEDANS. Subsequent incubation with DTT was performed to cleave off possible covalently attached persulfides as 1,5-AEDANS-sulfide conjugates (*i.e.* persulfurated proteins would finally not be visible under UV light). The covalently attached protein-thiosulfonate groups are not reactive *per se* with 1,5-I-AEDANS. As evident from Fig. 3, DsrE2B was uniformly 1,5-AEDANS-labeled irrespective of pretreatment, implying that the protein did not react with any of the tested compounds *in vitro*. DsrE3A and TusA were not modified by NaSH, GSSH, polysulfide, thiosulfate, or sulfite, but they reacted with tetrathionate. As shown in Fig. 3, fluorescence was not seen for tetrathionate-incubated DsrE3A and was substantially lower than after treatment with the other tested sulfur group donors for TusA. The residual fluorescence of tetrathionate-treated TusA might be either due to partial reactivity of TusA or a greater susceptibility of the TusA-Cys^18^-*S*-thiosulfate to hydrolysis. Control experiments showed that the tetrathionate-treated proteins were *a priori* unable to react with 1,5-I-AEDANS, pointing to a modification of cysteines with sulfonate or *S*-thiosulfonate rather than sulfane groups. This conclusion was verified by an independent experimental approach. Mass changes arising after incubation with the different tested sulfur compounds were analyzed by MALDI-TOF mass spectrometry (Fig. 4). Upon incubation with tetrathionate, DsrE3A gained a mass of 226 Da, which corresponded to a tetrathionate group or two thiosulfate groups (Fig. 4, *A* and *B*). The protein harbors two conserved cysteine residues, Cys^93^ and Cys^101^. Mutants DsrE3A-C^93^S (DsrE3A-Cys^101^) and DsrE3A-C^101^S (DsrE3A-Cys^93^) retained the ability to interact with tetrathionate, and each mutant protein covalently attached a group of 112 ± 4 Da, matching the molecular mass of thiosulfate (Table 2). The DsrE3A-C^93^S/C^101^S derivative lacking Cys^93^ as well as Cys^101^ stayed unmodified after incubation with tetrathionate (Table 2). Thus, both cysteine residues, Cys^93^ and Cys^101^, are individually and independently modified by attachment of a thiosulfate group upon incubation with tetrathionate as shown in Scheme 1.

**TABLE 2:** **MALDI-TOF MS detection of protein molecular masses changes after incubation with tetrathionate** Numbers in parentheses represent mass increases. NM, no modification.

Proteins	Theoretical molecular mass	Observed molecular mass	Expected modifications
	*Da*	*Da*	
DsrE3A	17,566	17,792 (226)	DsrE3A-Cys-*S*- (*S*-SO_3_)_2_
DsrE3A-C^93^S	17,550	17,658 (108)	DsrE3A-Cys^101^-*S-S*-SO_3_
DsrE3A-C^101^S	17,550	17,660 (110)	DsrE3A-Cys^93^-*S-S*-SO_3_
DsrE3A-(C^93^S/C^101^S)	17,534	17,533	NM
TusA	10,764	10,874 (110)	TusA-Cys^18^-*S-S*-SO_3_
TusA-C^18^S	10,748	10,748	NM

TusA from *M. cuprina* was also proven by mass spectrometry to form a Cys-*S*-thiosulfonate derivative upon incubation with tetrathionate (Fig. 4, *C* and *D*). A mass increase of 111 Da suggested that TusA also covalently attached a thiosulfate group. Because the mutant protein TusA-C^18^S lost the ability to react with the tetrathionate, it is deduced that the conserved cysteine residue of TusA played a key role in the reaction with tetrathionate (Table 2).

##### DsrE3A-Cys-S-Thiosulfonate Transfers a Thiosulfate Group to TusA

Our previous experiments described above established that DsrE3A and TusA were capable of mobilizing thiosulfate from tetrathionate. In the next step we set out to investigate whether DsrE3A-Cys-*S*-thiosulfonate could serve as a thiosulfate donor for TusA.

**FIGURE 5.**
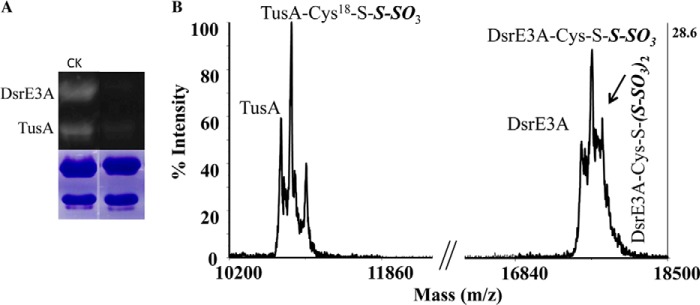
**Detection of thiosulfate transfer from DsrE3A-Cys-*S*-thiosulfonate to TusA by 1,5-I-AEDANS gel assay (*A*) and MALDI-TOF MS (*B*).** In *B*, an unidentified peak at 11,033 *m*/*z* was observed; this does not correspond to any second modification of TusA by thiosulfate. *CK*, represents a test with free DsrE3A and TusA. 2 nmol of DsrE3A or DsrE3A-Cys-*S*-thiosulfonate was loaded to the respective lanes.

DsrE3A-Cys-*S*-thiosulfonate and TusA were mixed at molar ratio of 3:2 and incubated at 65 °C for 30 min. Visualization of TusA with 1,5-I-AEDANS was not successful (Fig. 5*A*). MALDI-TOF MS confirmed thiosulfate transfer from DsrE3A-Cys-*S*-thiosulfonate to TusA (Fig. 5*B*). Not only were the peaks representing TusA and DsrE3A-Cys-*S*-thiosulfonate observed, but a newly emergent peak matched the mass of TusA-Cys^18^-*S*-thiosulfonate, which was deduced to be a product resulting from the transfer of a thiosulfate group from DsrE3A-Cys-*S*-thiosulfonate to TusA.

Mutants of DsrE3A were also assayed. The results (Table 3) showed that the mutated protein carrying a single replacement of cysteine residues, *i.e.* DsrE3A-C^93^S or DsrE3A-C^101^S, retained its ability to transfer thiosulfate to TusA.

**TABLE 3:** **Detection of thiosulfate transfer between DsrE3A and TusA** Numbers in parentheses represent mass increases. NM, no modification.

Proteins	Observed molecular mass	Expected modifications
	*Da*	
DsrE3A-Cys^101^-*S-S*-SO_3_ and TusA	10,873 (109)	TusA-Cys^18^-*S-S*-SO_3_
	17,659 (109)	DsrE3A-Cys^101^-*S-S*-SO_3_
DsrE3A-Cys^93^-*S-S*-SO_3_ and TusA	10,874 (110)	TusA-Cys^18^-*S-S*-SO_3_
	17,658 (108)	DsrE3A-Cys^93^-*S-S*-SO_3_
DsrE3A-(C^93^S/C^101^S) and TusA	10,765	NM
	17,533	NM
DsrE3A-Cys-*S*-(*S*-SO_3_)_2_ and TusA-C^18^S	10,747	NM
	17,790 (224)	DsrE3A-Cys-*S*- (*S*-SO_3_)_2_
TusA-Cys^18^-*S-S*-SO_3_ and DsrE3A	10,763	NM
	17,564	NM
TusA-Cys^18^-*S-S*-SO_3_ and DsrE3A-C^93^S	10,763	NM
	17,554	NM
TusA-Cys^18^-*S-S*-SO_3_ and DsrE3A-C^101^S	10,764	NM
	17,553	NM
TusA-Cys^18^-*S-S*-SO_3_ and DsrE3A-(C^93^S/C^101^S)	10,877 (113)	TusA-Cys^18^-*S-S*-SO_3_
	17,533	NM

##### TusA-Cys^18^-S-Thiosulfonate Does Not Transfer Thiosulfate to DsrE3A, and DsrE3A Cleaves TusA-Cys^18^-S-Thiosulfonate

**FIGURE 6.**
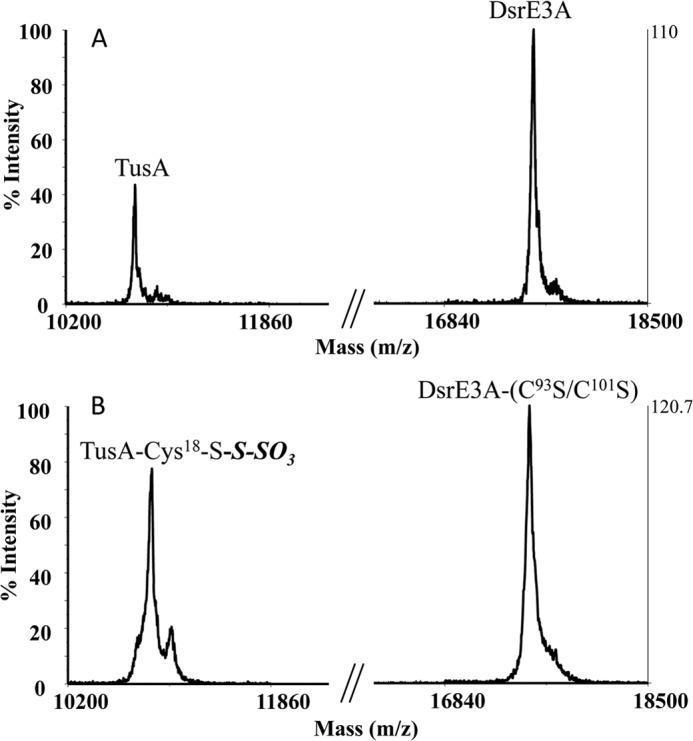
**DsrE3A cleaved the thiosulfate group from TusA-Cys^18^-*S*-thiosulfonate (*A*), but DsrE3A-C^93^S/C^101^S was not able to do so (*B*).**

We further determined whether TusA-Cys^18^-*S*-thiosulfonate was able to transfer its thiosulfate group to DsrE3A. The thiosulfate group of TusA-Cys^18^-*S*-thiosulfonate did not transfer to DsrE3A, but no trace of it was detected in the MALDI mass spectrum after incubation (Fig. 6*A*). Thus, we deduced that DsrE3A cleaved the TusA-Cys^18^-*S*-thiosulfonate and released free TusA. Additional experiments (Fig. 6*B*) showed that the double replacement mutant DsrE3A-C^93^S/C^101^S lost the ability to cleave the thiosulfate group from TusA-Cys^18^-*S*-thiosulfonate, suggesting that cysteine residues of DsrE3A played an important role during cleavage.

We also tested whether DsrE3A-Cys-*S*-thiosulfonate and TusA-Cys^18^-*S*-thiosulfonate were able to transfer their thiosulfate groups to DsrE2B. Thiosulfate transfer to DsrE2B was not observed (data not shown).

##### DsrE3A and TusA Interact Physically with Each Other and Form a Heterocomplex

**FIGURE 7.**
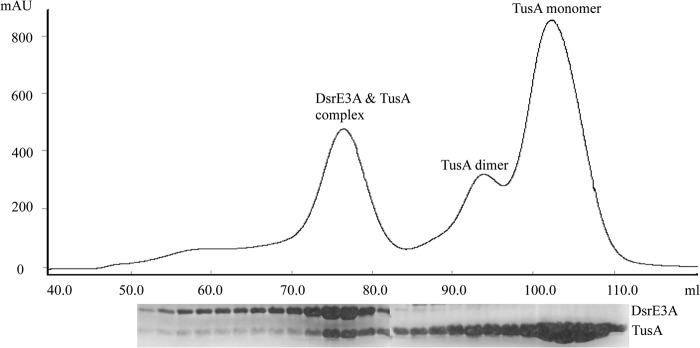
**DsrE3A and TusA form a heterocomplex.** The protein components of the fractions were determined with SDS-PAGE, and the results are shown below the elution curve.

As demonstrated above, DsrE3A and TusA both reacted with tetrathionate resulting in DsrE3A-Cys-*S*-thiosulfonate and TusA-Cys^18^-*S*-thiosulfonate. In addition, DsrE3A cleaved TusA-Cys^18^-*S*-thiosulfonate. These results invoked the idea that DsrE3A and TusA might interact physically with each other. Pulldown assays were conducted for DsrE3A plus Strep-tagged TusA with Strep-Tactin Superflow gravity flow columns. In a control experiment, DsrE3A did not bind to the affinity matrix, whereas a considerable portion was retained on the column in the presence of Strep-tagged TusA and co-eluted with it (data not shown). DsrE3A did not interact with TusA-C^18^S, in which the active site cysteine was replaced by serine, showing that Cys^18^ of TusA is indispensable for the interaction. Analysis by gel permeation chromatography showed that DsrE3A and TusA formed a heterocomplex after they were incubated together (Fig. 7). These results suggested a specific interaction between DsrE3A and TusA. Using the same methods, interactions were not observed between DsrE2B and TusA or DsrE3A.

**FIGURE 8.**
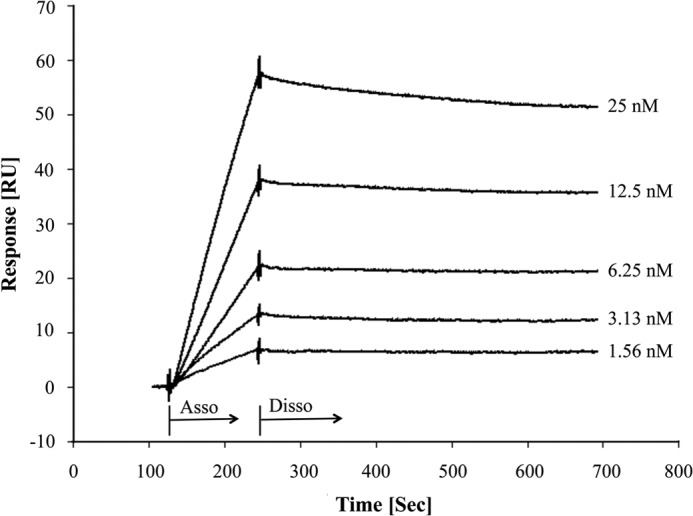
**Determination of association and dissociation between DsrE3A and TusA by surface plasmon resonance.** *RU*, resonance unit.

Moreover, surface plasmon resonance was applied to quantitatively evaluate the affinity between DsrE3A and TusA (Fig. 8). DsrE3A was covalently immobilized on a CM5 sensor chip. TusA at different concentrations flowed over the chip surface. The equilibrium dissociation constant (*K_D_*) according to the fitted model was 0.1 nm. The very low value of *K_D_* demonstrated a strong interaction between DsrE3A and TusA.

## DISCUSSION

**FIGURE 9.**
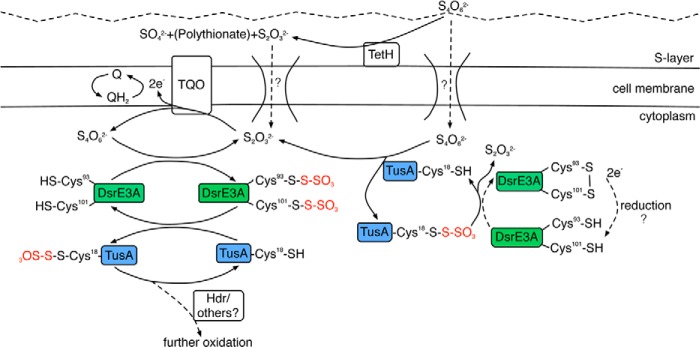
**Proposed roles of DsrE3A and TusA in dissimilatory sulfur metabolism in *M. cuprina*.** Systems for transport of thiosulfate and tetrathionate into the cytoplasm have thus far not been identified in *M. cuprina. TetH*, tetrathionate hydrolase.

This work demonstrated that proteins DsrE3A and TusA from the acidothermophilic archaeon *M. cuprina* Ar-4 have the ability to mobilize thiosulfate from tetrathionate. Moreover, thiosulfate transfer from DsrE3A-Cys-*S*-thiosulfonate to TusA was shown. To our knowledge, proteins that react with tetrathionate and form protein-Cys-*S*-thiosulfonates have thus far not been reported in sulfur oxidizers. Thus, DsrE3A and TusA from *M. cuprina* Ar-4 represent the first pair of proteins with such novel properties. Although both DsrE3A and TusA were able to react with tetrathionate, we observed that DsrE3A-Cys-*S*-thiosulfonate further transferred its thiosulfate group to TusA. This observation might imply that DsrE3A functions as a thiosulfate donor to TusA *in vivo*. Thiosulfonated TusA could then serve as the substrate for other enzymes such as the *hdrC1B1AhyphdrC2B2*-encoded proteins (Fig. 9). We envision that the sulfonate group is first released, either hydrolytically as sulfate or reductively as sulfite, by an as yet unknown mechanism. The sulfane group remaining on TusA could then be oxidized and finally also released.

The close genomic linkage of the TusA-encoding gene (Mcup_0683) with *hdr*-like genes and the DsrE3A-encoding gene (Mcup_0681) with a lipoamide dehydrogenase-encoding gene, not only in *M. cuprina* but also in other sulfur-oxidizing archaea and bacteria (Figs. 1 and 2*B*), opens the possibility of a functional linkage of these systems. This idea is corroborated by the tight interaction of archaeal TusA and DsrE3A proven in this work by several independent experimental approaches. Involvement of a lipoamide-binding protein as a potential sulfur carrier and linkage to lipoamide dehydrogenase could even result in transfer of some of the electrons arising from sulfane sulfur oxidation to NAD^+^.

In addition to the reaction with tetrathionate that leads to binding of a thiosulfonate group, we showed that DsrE3A is able to release thiosulfate from TusA-Cys^18^-*S*-thiosulfonate. This reaction requires two electrons. In principle, such a mechanism resembles the reverse of the oxidative binding of thiosulfate to a cysteine of the SoxYZ protein, which occurs as the first step of thiosulfate oxidation catalyzed by the Sox system (42). We envision that the electrons required stem from the formation of an intramolecular disulfide between the two conserved cysteine residues of DsrE3A or from the formation of an intermolecular disulfide between two molecules of DsrE3A. The formation of an intermolecular disulfide is supported by the observation that single cysteine replacement mutants DsrE3A-C^93^S and DsrE3A-C^101^S retained the ability to release thiosulfate from TusA-Cys^18^-*S*-thiosulfonate.

Our results demonstrated that TusA is involved in thiosulfate transfer. Based on the finding that TusA reacts with tetrathionate and that DsrE3A cleaves a thiosulfate group from TusA-Cys^18^-*S*-thiosulfonate, we propose that TusA is involved in dissimilatory oxidation of tetrathionate in *M. cuprina* Ar-4 when grown with tetrathionate as energy source, a role distinct from the function of TusA as a sulfurtransferase in tRNA modification (43) and molybdenum cofactor biosynthesis (44) in *E. coli*. TusA of *M. cuprina* Ar-4 functions as a dissimilatory protein, just as has been reported for TusA from the purple sulfur bacterium *A. vinosum* (24).

As an alternative function, or even in addition to the model elaborated above, archaeal TusA could generate thiosulfate in the cytoplasm, which would be further oxidized by TQO (Fig. 9). TQO oxidizes thiosulfate and transfers electrons via caldariellaquinone (14). Genes coding for TQO are present in the genome of *M. cuprina* Ar-4 (11), and the active site of TQO in *A. ambivalens* has been suggested to face toward the cytoplasm (45). Tetrathionate produced by TQO is thus released to the cytoplasm, where DsrE3A is located, and further converts tetrathionate (Fig. 9).

## Footnotes

5 The abbreviations used are:

TQO: thiosulfate:quinone oxidoreductase
1,5-I-AEDANS: *N*-(iodoacetyl)-*N*′-(5-sulfo-1-naphthyl)ethylenediamine
PDB: Protein Data Bank.

## References

[1] Waksman S. A. Joffe J. S. (1922) Microorganisms concerned in the oxidation of sulfur in the soil. II. *Thiobacillus thiooxidans*, a new sulfur-oxidizing organism isolated from the soil. J. Bacteriol. 7, 239–256 16558952 10.1128/jb.7.2.239-256.1922 PMC378965

[2] Dopson M. Johnson D. B. (2012) Biodiversity, metabolism and applications of acidophilic sulfur-metabolizing microorganisms. Environ. Microbiol. 14, 2620–2631 22510111 10.1111/j.1462-2920.2012.02749.x

[3] Rohwerder T. Sand W. (2007) Oxidation of inorganic sulfur compounds in acidophilic prokaryotes. Eng. Life Sci. 7, 301–309

[4] Imhoff J. F. Süling J. Petri R. (1998) Phylogenetic relationships among the Chromatiaceae, their taxonomic reclassification and description of the new genera *Allochromatium, Halochromatium, Isochromatium, Marichromatium, Thiococcus, Thiohalocapsa,* and *Thermochromatium*. Int. J. Syst. Bacteriol. 48, 1129–1143 9828415 10.1099/00207713-48-4-1129

[5] Huber G. Spinnler C. Gambacorta A. Stetter K. O. (1989) *Metallosphaera sedula* gen. and sp. nov. represents a new genus of aerobic, metal-mobilizing, thermoacidophilic archaebacteria. Syst. Appl. Microbiol. 12, 38–47

[6] Liu L. J. You X. Y. Guo X. Liu S. J. Jiang C. Y. (2011) *Metallosphaera cuprina* sp. nov., an acidothermophilic, metal-mobilizing archaeon. Int. J. Syst. Evol. Microbiol. 61, 2395–2400 21057050 10.1099/ijs.0.026591-0

[7] Kletzin A. (1989) Coupled enzymatic production of sulfite, thiosulfate, and hydrogen sulfide from sulfur: purification and properties of a sulfur oxygenase reductase from the facultatively anaerobic archaebacterium *Desulfurolobus ambivalens*. J. Bacteriol. 171, 1638–1643 2493451 10.1128/jb.171.3.1638-1643.1989 PMC209792

[8] Sun C. W. Chen Z. W. He Z. G. Zhou P. J. Liu S. J. (2003) Purification and properties of the sulfur oxygenase/reductase from the acidothermophilic archaeon, *Acidianus* strain S5. Extremophiles 7, 131–134 12664265 10.1007/s00792-002-0304-5

[9] Chen Z. W. Liu Y. Y. Wu J. F. She Q. Jiang C. Y. Liu S. J. (2007) Novel bacterial sulfur oxygenase reductases from bioreactors treating gold-bearing concentrates. Appl. Microbiol. Biotechnol. 74, 688–698 17111141 10.1007/s00253-006-0691-0

[10] Auernik K. S. Maezato Y. Blum P. H. Kelly R. M. (2008) The genome sequence of the metal-mobilizing, extremely thermoacidophilic archaeon *Metallosphaera sedula* provides insights into bioleaching-associated metabolism. Appl. Environ. Microbiol. 74, 682–692 18083856 10.1128/AEM.02019-07 PMC2227735

[11] Liu L. J. You X. Y. Zheng H. Wang S. Jiang C. Y. Liu S. J. (2011) Complete genome sequence of *Metallosphaera cuprina*, a metal sulfide-oxidizing archaeon from a hot spring. J. Bacteriol. 193, 3387–3388 21551305 10.1128/JB.05038-11 PMC3133273

[12] Friedrich C. G. Rother D. Bardischewsky F. Quentmeier A. Fischer J. (2001) Oxidation of reduced inorganic sulfur compounds by bacteria: emergence of a common mechanism? Appl. Environ. Microbiol. 67, 2873–2882 11425697 10.1128/AEM.67.7.2873-2882.2001 PMC92956

[13] Ghosh W. Dam B. (2009) Biochemistry and molecular biology of lithotrophic sulfur oxidation by taxonomically and ecologically diverse bacteria and archaea. FEMS Microbiol. Rev. 33, 999–1043 19645821 10.1111/j.1574-6976.2009.00187.x

[14] Müller F. H. Bandeiras T. M. Urich T. Teixeira M. Gomes C. M. Kletzin A. (2004) Coupling of the pathway of sulphur oxidation to dioxygen reduction: characterization of a novel membrane-bound thiosulphate: quinone oxidoreductase. Mol. Microbiol. 53, 1147–1160 15306018 10.1111/j.1365-2958.2004.04193.x

[15] Denkmann K. Grein F. Zigann R. Siemen A. Bergmann J. van Helmont S. Nicolai A. Pereira I. A. Dahl C. (2012) Thiosulfate dehydrogenase: a widespread unusual acidophilic *c*-type cytochrome. Environ. Microbiol. 14, 2673–2688 22779704 10.1111/j.1462-2920.2012.02820.x

[16] Liu Y. W. Denkmann K. Kosciow K. Dahl C. Kelly D. J. (2013) Tetrathionate stimulated growth of *Campylobacter jejuni* identifies a new type of bi-functional tetrathionate reductase (TsdA) that is widely distributed in bacteria. Mol. Microbiol. 88, 173–188 23421726 10.1111/mmi.12176

[17] Brito J. A. Sousa F. L. Stelter M. Bandeiras T. M. Vonrhein C. Teixeira M. Pereira M. M. Archer M. (2009) Structural and functional insights into sulfide: quinone oxidoreductase. Biochemistry 48, 5613–5622 19438211 10.1021/bi9003827

[18] Pott A. S. Dahl C. (1998) Sirohaem sulfite reductase and other proteins encoded by genes at the *dsr* locus of *Chromatium vinosum* are involved in the oxidation of intracellular sulfur. Microbiology 144, 1881–1894 9695921 10.1099/00221287-144-7-1881

[19] Berg J. S. Schwedt A. Kreutzmann A. C. Kuypers M. M. Milucka J. (2014) Polysulfides as intermediates in the oxidation of sulfide to sulfate by *Beggiatoa* spp. Appl. Environ. Microbiol. 80, 629–636 24212585 10.1128/AEM.02852-13 PMC3911116

[20] Dahl C. Engels S. Pott-Sperling A. S. Schulte A. Sander J. Lübbe Y. Deuster O. Brune D. C. (2005) Novel genes of the *dsr* gene cluster and evidence for close interaction of Dsr proteins during sulfur oxidation in the phototrophic sulfur bacterium *Allochromatium vinosum*. J. Bacteriol. 187, 1392–1404 15687204 10.1128/JB.187.4.1392-1404.2005 PMC545617

[21] Lübbe Y. J. Youn H. S. Timkovich R. Dahl C. (2006) Siro (haem) amide in *Allochromatium vinosum* and relevance of DsrL and DsrN, a homolog of cobyrinic acid a, *c*-diamide synthase, for sulphur oxidation. FEMS Microbiol. Lett. 261, 194–202 16907720 10.1111/j.1574-6968.2006.00343.x

[22] Sander J. Engels-Schwarzlose S. Dahl C. (2006) Importance of the DsrMKJOP complex for sulfur oxidation in *Allochromatium vinosum* and phylogenetic analysis of related complexes in other prokaryotes. Arch. Microbiol. 186, 357–366 16924482 10.1007/s00203-006-0156-y

[23] Stockdreher Y. Venceslau S. S. Josten M. Sahl H. G. Pereira I. A. Dahl C. (2012) Cytoplasmic sulfurtransferases in the purple sulfur bacterium *Allochromatium vinosum*: evidence for sulfur transfer from DsrEFH to DsrC. PloS One 7, e40785 22815818 10.1371/journal.pone.0040785 PMC3397948

[24] Stockdreher Y. Sturm M. Josten M. Sahl H. G. Dobler N. Zigann R. Dahl C. (2014) New proteins involved in sulfur trafficking in the cytoplasm of *Allochromatium vinosum*. J. Biol. Chem. 289, 12390–12403 24648525 10.1074/jbc.M113.536425 PMC4007435

[25] Quatrini R. Appia-Ayme C. Denis Y. Jedlicki E. Holmes D. S. Bonnefoy V. (2009) Extending the models for iron and sulfur oxidation in the extreme acidophile *Acidithiobacillus ferrooxidans*. BMC Genomics 10, 394 19703284 10.1186/1471-2164-10-394 PMC2754497

[26] You X. Y. Liu C. Wang S. Y. Jiang C. Y. Shah S. A. Prangishvili D. She Q. Liu S. J. Garrett R. A. (2011) Genomic analysis of *Acidianus hospitalis* W1, a host for studying crenarchaeal virus and plasmid life cycles. Extremophiles 15, 487–497 21607549 10.1007/s00792-011-0379-y PMC3119797

[27] Kawarabayasi Y. Hino Y. Horikawa H. Jin-no K. Takahashi M. Sekine M. Baba S. Ankai A. Kosugi H. Hosoyama A. Fukui S. Nagai Y. Nishijima K. Otsuka R. Nakazawa H. Takamiya M. Kato Y. Yoshizawa T. Tanaka T. Kudoh Y. Yamazaki J. Kushida N. Oguchi A. Aoki K. Masuda S. Yanagii M. Nishimura M. Yamagishi A. Oshima T. Kikuchi H. (2001) Complete genome sequence of an aerobic thermoacidophilic crenarchaeon, *Sulfolobus tokodaii* strain7. DNA Res. 8, 123–140 11572479 10.1093/dnares/8.4.123

[28] Auernik K. S. Kelly R. M. (2008) Identification of components of electron transport chains in the extremely thermoacidophilic crenarchaeon *Metallosphaera sedula* through iron and sulfur compound oxidation transcriptomes. Appl. Environ. Microbiol. 74, 7723–7732 18931292 10.1128/AEM.01545-08 PMC2607173

[29] Deckert G. Warren P. V. Gaasterland T. Young W. G. Lenox A. L. Graham D. E. Overbeek R. Snead M. A. Keller M. Aujay M. (1998) The complete genome of the hyperthermophilic bacterium *Aquifex aeolicus*. Nature 392, 353–358 9537320 10.1038/32831

[30] Reysenbach A. L. Hamamura N. Podar M. Griffiths E. Ferreira S. Hochstein R. Heidelberg J. Johnson J. Mead D. Pohorille A. (2009) Complete and draft genome sequences of six members of the *Aquificales*. J. Bacteriol. 191, 1992–1993 19136599 10.1128/JB.01645-08 PMC2648382

[31] You X. Y. Guo X. Zheng H. J. Zhang M. J. Liu L. J. Zhu Y. Q. Zhu B. Wang S. Y. Zhao G. P. Poetsch A. (2011) Unraveling the *Acidithiobacillus caldus* complete genome and its central metabolisms for carbon assimilation. J. Genet. Genomics 38, 243–252 21703548 10.1016/j.jgg.2011.04.006

[32] Muyzer G. Sorokin D. Y. Mavromatis K. Lapidus A. Foster B. Sun H. Ivanova N. Pati A. D'haeseleer P. Woyke T. Kyrpides N. C. (2011) Complete genome sequence of *Thioalkalivibrio* sp. K90mix. Stand. Genomic Sci. 5, 341–355 22675584 10.4056/sigs.2315092 PMC3368412

[33] Brock T. D. Brock K. M. Belly R. T. Weiss R. L. (1972) *Sulfolobus*: a new genus of sulfur-oxidizing bacteria living at low pH and high temperature. Arch. Mikrobiol. 84, 54–68 4559703 10.1007/BF00408082

[34] Horton R. M. (1995) PCR-mediated recombination and mutagenesis: SOEing together tailor-made genes. Mol. Biotechnol. 3, 93–99 7620981 10.1007/BF02789105

[35] Tamura K. Stecher G. Peterson D. Filipski A. Kumar S. (2013) MEGA6: Molecular Evolutionary Genetics Analysis version 6.0. Mol. Biol. Evol. 30, 2725–2729 24132122 10.1093/molbev/mst197 PMC3840312

[36] Sneath P. H. A. Sokal R. R. (1973) Numerical Taxonomy, the Principles and Practice of Numerical Classification. W. H. Freeman, San Francisco

[37] Felsenstein J. (1985) Confidence limits on phylogenies: an approach using bootstrap. Evolution 39, 783–791 10.1111/j.1558-5646.1985.tb00420.x 28561359

[38] Zuckerkandl E. Pauling L. (1965) Evolutionary divergence and convergence in proteins, in Evolving Genes and Proteins (Bryson V. Vogel H. J., eds) pp. 97–166, Academic Press, New York

[39] Zheng L. White R. H. Cash V. L. Dean D. R. (1994) Mechanism for the desulfurization of l-cysteine catalyzed by the nifS gene product. Biochemistry 33, 4714–4720 8161529 10.1021/bi00181a031

[40] Rohwerder T. Sand W. (2003) The sulfane sulfur of persulfides is the actual substrate of the sulfur-oxidizing enzymes from *Acidithiobacillus* and *Acidiphilium* spp. Microbiology 149, 1699–1710 12855721 10.1099/mic.0.26212-0

[41] Thomé R. Gust A. Toci R. Mendel R. Bittner F. Magalon A. Walburger A. (2012) A sulfurtransferase is essential for activity of formate dehydrogenases in *Escherichia coli*. J. Biol. Chem. 287, 4671–4678 22194618 10.1074/jbc.M111.327122 PMC3281601

[42] Friedrich C. G. Quentmeier A. Bardischewsky F. Rother D. Orawski G. Hellwig P. Fischer J. (2008) Redox control of chemotrophic sulfur oxidation of *Paracoccus pantotrophus*, in Microbial Sulfur Metabolism (Dahl C. Friedrich C. G., eds) pp. 139–150, Springer-Verlag, Berlin Heidelberg

[43] Ikeuchi Y. Shigi N. Kato J. Nishimura A. Suzuki T. (2006) Mechanistic insights into sulfur relay by multiple sulfur mediators involved in thiouridine biosynthesis at tRNA wobble positions. Mol. Cell 21, 97–108 16387657 10.1016/j.molcel.2005.11.001

[44] Dahl J. U. Radon C. Bühning M. Nimtz M. Leichert L. I. Denis Y. Jourlin-Castelli C. Iobbi-Nivol C. Méjean V. Leimkühler S. (2013) The sulfur carrier protein TusA has a pleiotropic role in *Escherichia coli* that also affects molybdenum cofactor biosynthesis. J. Biol. Chem. 288, 5426–5442 23281480 10.1074/jbc.M112.431569 PMC3581435

[45] Protze J. Müller F. Lauber K. Naß B. Mentele R. Lottspeich F. Kletzin A. (2011) An extracellular tetrathionate hydrolase from the thermoacidophilic archaeon *Acidianus ambivalens* with an activity optimum at pH 1. Front. Microbiol. 2, 68 21747790 10.3389/fmicb.2011.00068 PMC3128947

[46] Venceslau S. S. Stockdreher Y. Dahl C. Pereira I. A. (2014) The “bacterial heterodisulfide” DsrC is a key protein in dissimilatory sulfur metabolism. Biochim. Biophys. Acta 1837, 1148–1164 24662917 10.1016/j.bbabio.2014.03.007

[47] Sherman F. Stewart J. W. Tsunasawa S. (1985) Methionine or not methionine at the beginning of a protein. BioEssays 3, 27–31 3024631 10.1002/bies.950030108

[48] Hirel P. H. Schmitter M. J. Dessen P. Fayat G. Blanquet S. (1989) Extent of N-terminal methionine excision from *Escherichia coli* proteins is governed by the side-chain length of the penultimate amino acid. Proc. Natl. Acad. Sci. U.S.A. 86, 8247–8251 2682640 10.1073/pnas.86.21.8247 PMC298257

[49] Hanahan D. (1983) Studies on transformation of *Escherichia coli* with plasmids. J. Mol. Biol. 166, 557–580 6345791 10.1016/s0022-2836(83)80284-8

